# Novel stereotactic body radiation therapy (SBRT)-based partial tumor irradiation targeting hypoxic segment of bulky tumors (SBRT-PATHY): improvement of the radiotherapy outcome by exploiting the bystander and abscopal effects

**DOI:** 10.1186/s13014-019-1227-y

**Published:** 2019-01-29

**Authors:** Slavisa Tubin, Helmut H. Popper, Luka Brcic

**Affiliations:** 1 KABEG Klinikum Klagenfurt, Institute of Radiation Oncology, Feschnigstraße 11, 9020 Klagenfurt am Wörthersee, Austria; 2 Medical University of Graz, Diagnostic and Research Institute of Pathology, Neue Stiftingtalstrasse 6, 8036 Graz, Austria

**Keywords:** Novel, SBRT, Partial, Bystander, Abscopal, Hypoxia, Bulky, Oligometastases

## Abstract

**Background:**

Despite the advances in oncology, patients with bulky tumors have worse prognosis and often receive only palliative treatments. Bulky disease represents an important challenging obstacle for all currently available radical treatment options including conventional radiotherapy. The purpose of this study was to assess a retrospective outcome on the use of a newly developed unconventional stereotactic body radiation therapy (**SBRT**) for **PA**rtial **T**umor irradiation of unresectable bulky tumors targeting exclusively their **HY**poxic segment (**SBRT-PATHY**) that exploits the non-targeted effects of radiotherapy: bystander effects (local) and the abscopal effects (distant).

**Materials and methods:**

Twenty-three patients with bulky tumors received partial bulky irradiation in order to induce the local non-targeted effect of radiation (bystander effect). The hypoxic tumor segment, called the bystander tumor volume (BTV), was defined using PET and contrast-enhanced CT, as a hypovascularized-hypometabolic junctional zone between the central necrotic and peripheral hypervascularized-hypermetabolic tumor segment. Based on tumor site and volume, the BTV was irradiated with 1–3 fractions of 10–12 Gy prescribed to 70% isodose-line. The pathologic lymph nodes and metastases were not irradiated in order to assess the distant non-targeted effects of radiation (abscopal effect). No patient received any systemic therapy.

**Results:**

At the time of analysis, with median follow-up of 9.4 months (range: 4–20), 87% of patients remained progression-free. The bystander and abscopal response rates were 96 and 52%, respectively. Median shrinkage of partially irradiated bulky tumor expressing intensity of the bystander effect was 70% (range 30–100%), whereas for the non-irradiated metastases (intensity of the abscopal effect), it was 50% (range 30–100%). No patient experienced acute or late toxicity of any grade.

**Conclusions:**

SBRT-PATHY showed very inspiring results on exploitation of the radiation-hypoxia-induced non-targeted effects that need to be confirmed through our ongoing prospective trial.

Present study has been retrospectively registered by the local ethic committee under study number A 26/18.

## Introduction

Despite the tremendous oncological developments, patients with bulky tumors still have poor prognosis, and often receive only palliative treatments [1–5]. Such “heavyweight” tumors, because of their high volume, very intimate relationship with surrounding OAR, and voluminous hypoxic compartments, are difficult to treat successfully with currently available treatments. Therefore, exploiting the tumoricidal non-targeted effects (NTE) of radiotherapy by harnessing the local bystander (BE) and the distant abscopal effects (AE) may potentially enhance the radiotherapy therapeutic ratio.

The incorporation of NTE in routine clinical practice is still scarce although several studies have proven their existence and their potential beneficial outcome [6]. These studies provided an immune based explanation to the NTE phenomenon and rebuked skepticism on the ability of radiotherapy to one lesion to induce tumor shrinkage in a distant non-irradiated lesion. At a certain radiation dose, radiation-induced tumor cells damage activates antitumor immune response through the release of tumor antigens and damage-associated molecular pattern, which, in turn, results in increased activation of antigen-presenting cells and T-lymphocytes. This activation of the immune system triggers antigen-specific, adaptive immunity, a phenomenon referred to as “in situ” radio-vaccination [6]. The recent understanding of cancer immunotherapy led to increased exploitation of AE-induction through utilization of combined radioimmunotherapy. However, the reason for the lack of full expression of this phenomenon is possibly related to the fact that the conventional radiotherapy is performed in conditions that do not favor AE manifestation.

In order to improve the role of radiotherapy as an inductor of NTE, we have previously proved for the first time that the irradiation of hypoxic tumor cells resulted in effective *radiation-hypoxia-induced bystander (R-H-IBE) and abscopal effects (R-H-IAE)* [7]. Hypoxic cells, due to their different tumor biology, showed a higher potential for NTE than the normoxic cells which, being more sensitive and mostly killed by higher radiation dose, exhibit weaker *abscopal tumor signaling*. While the hypoxia-induced increase in secretion of vascular endothelial growth factor, basic fibroblast growth factor, and placenta growth factor is known to promote tumor growth and progression [8–10], an expression of anti-angiogenic, fms-like tyrosine kinase (Flt-1) is shown to be downregulated by hypoxia [11]. In our previous study [7], the levels of Flt-1 correlated with the observed R-H-IAE. Our pre-clinical findings were translated to the clinic, and new unconventional *SBRT* for *PA*rtial *T*umor irradiation targeting *HY*poxic segment *(SBRT-PATHY)* was developed [12]. Our study aim to assess the role of this novel approach, exploiting BE and AE, in improving radiotherapy outcomes.

**Fig. 1:**
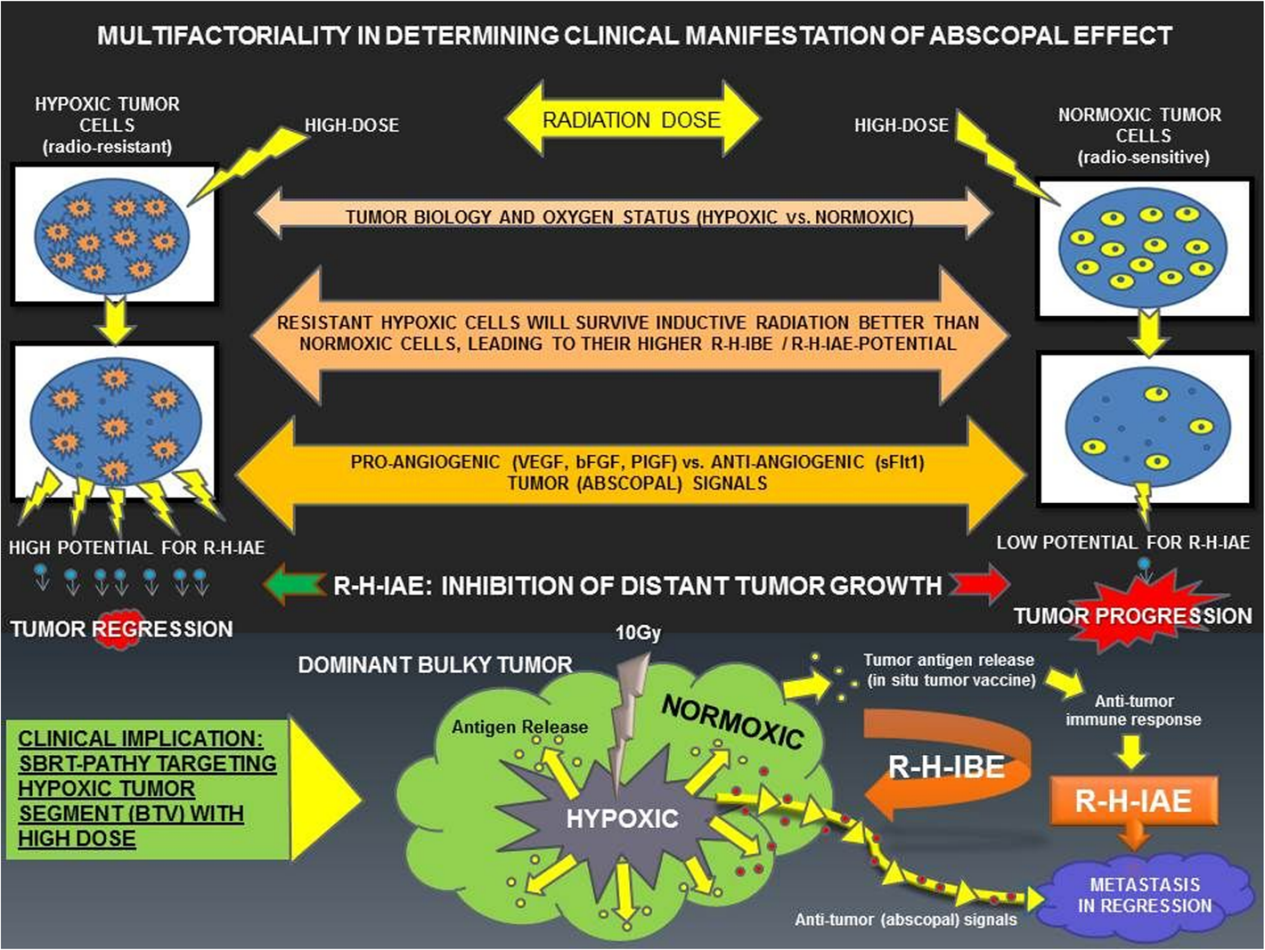
HYPOTHESIS; RADIATION-HYPOXIA-INDUCED BYSTANDER (BE) AND ABSCOPAL EFFECTS (AE): The hypoxic tumor cells showed higher “abscopal potential” than did the normoxic tumor cells, probably due to their higher survival following inductive radiation (10 Gy). In addition to the differential radio-sensitivity, the definitive BE/AE-intensity is determined by multiple factors, such as radiation dose, tumor biology, oxygen status, and balance between pro-angiogenic and anti-angiogenic “abscopal messengers.” The lower part of the figure shows the radiobiology of SBRT-PATHY: irradiation of hypoxic segment results in induction of radiation-hypoxia-induced BE and AE, leading to regression of partially irradiated tumor as well as unirradiated distant metastases

In this retrospective analysis, we report on the use of SBRT-PATHY applied to very difficult clinical situations for the treatment of unresectable bulky tumors, most of which were metastatic. We tested the validity of our hypothesis (Fig. 1) that high-dose irradiation of hypoxic bulky segment would generate clinically significant regression of partially irradiated bulky (due to R-H-IBE) but also of unirradiated metastases (due to R-H-IAE). The primary endpoints were BE and AE response rates. The secondary endpoints included overall survival, progression-free survival, safety, and SBRT-PATHY’s neoadjuvant potential in converting unresectable bulky tumors into resectable lesions.

## Material and methods

### Translation of pre-clinical findings to the clinic

Pre-clinical phase of this translational research confirmed that hypoxic and normoxic tumor compartments, if selectively irradiated as different “inductors” of NTE, could generate NTE of different intensities [7]. Because the hypoxic tumor compartment was responsible for the stronger NTE after high-dose irradiation (10Gy × 1), we selected irradiation of the hypoxic tumor segment (as inductor of NTE) and high fraction-dose radiotherapy as factors that could predict for induction of BE/AE. Subsequent translation of these findings to a clinical setting resulted in the development of a novel partial high-dose tumor irradiation targeting the hypoxic tumor segment [12].

### Target population

Male or female patients older than 18 years undergoing SBRT-PATHY for unresectable solid “bulky” malignancies located in the chest, the abdomen or the head and neck area, with limited treatment options, and who are ineligible for or are in progression under the systemic therapy (Table 1). “Bulky” disease was considered as a substantial, unresectable tumor mass larger than 6 cm on CT scan at diagnosis.

**Table 1:** Patient and treatment characteristics for patients with unresectable bulky tumors treated with SBRT partial tumor irradiation targeting the hypoxic tumor segment (SBRT-PATHY)

Characteristic	No. (%)
Sex	
Male	17 (74)
Female	6 (26)
Median age at SBRT-PATHY	74 years, range: 41–86
Primary tumor site	
Lung	16 (69.6)
Kidney	3 (13.1)
Skin	2 (8.7)
Prostate	1 (4.3)
Unknown	1 (4.3)
Histology	
Adenocarcinoma	5 (21.7)
Squamous	13 (56.6)
Melanoma	1 (4.3)
Renal cell carcinoma	3 (13.1)
Unknown	1 (4.3)
Treated site	
Lymph node metastases: Neck levels IIA right and IV left Retroperitoneal Mediastinum	5 (21.7)
Lung primary: Central: ULL, LLL, URL, Mediastinum Peripheral: LRL, URL, ULL, LLL	14 (60.9)
Lung metastases: Peripheral: LRL, ULL	3 (13.1)
Adrenal gland (left)	1 (4.3)
Oligo-metastatic patients:	14 (60.9)
1 metastasis	3 (21.4)
> 1 ≤ 3 metastases	8 (57.1)
> 3 ≤ 5 metastases	3 (21.4)
Metastatic site(s)	Lung, Neck, Mediastinum, Soft Tissue, Retroperitoneal, Adrenal, Bone, Liver
Metastases in the same organ as treated bulky	2 (14.3)
Metastases in the different organ(s)	7 (50)
Both (in the same and in the different organs)	5 (35.7)
Systemic therapy	7 (30.4)
Chemotherapy	4 (17)
Immunotherapy	2 (8.7)
Hormonal (androgen deprivation therapy)	1 (4.3)
GTV volume mean/range (cm^3^)	179.8/30–499
GTV diameter mean/range (cm)	9.1/6–17
BTV volume mean/range (cm^3^)	57.1/5–137
SUVmax mean/range for GTV	23/7–85
SUVmax mean/range for BTV	3/1–3
Prescription dose	
10Gy × 1 to 70% isodose-line	15 (65.2)
12Gy × 1 to 70% isodose-line	4 (17.4)
10Gy × 3 to 70% isodose-line	4 (17.4)

Abbreviations: ULL-upper left lobe, LLL-lower left lobe, URL-upper right lobe, LRL-lower right lobe, GTV-gross tumor volume, BTV-bystander tumor volume, SBRT-PATHY-SBRT PArtial Tumor irradiation of the HYpoxic segment, SUV-standardized uptake values

### Target definition

**Fig. 2:**
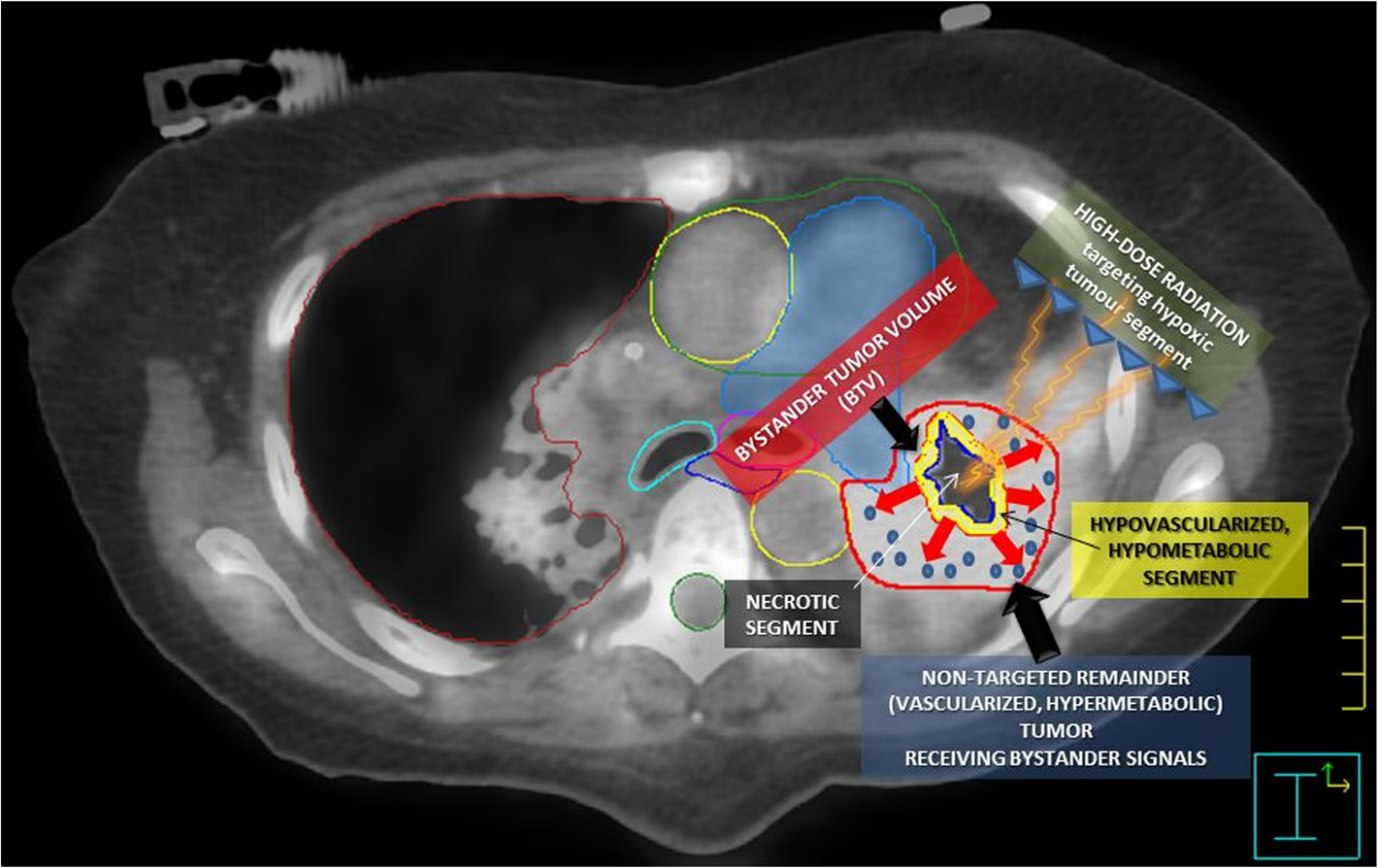
DEFINITION OF THE BYSTANDER TUMOR VOLUME (BTV): The figure summarizes the radiobiology of the bystander effect-induction by SBRT-PATHY. An 18F-FDG PET combined with a contrast-enhanced CT was used for the definition of BTV (smaller yellow contour), which corresponds to the junctional region between the central necrotic segment (black region) and the contrast-enhanced, hypermetabolic peripheral tumor (red contour, not targeted for irradiation). The red arrows represent “anti-angiogenic bystander signal” (blue pellets) released by the irradiated hypoxic tumor, inducing the regression of the non-targeted tumor

After a simulation-CT was fused with 18F-FDG PET and contrast enhanced CT, we divided each bulky tumor into three segments (Fig. 2) [12]:

- contrast-enhanced (vascularized) peripheral tumor segment,
- contrast-unenhanced (necrotic) central tumor segment,
- *contrast-hypoenhanced* (*hypovascularized*) tumor segment as an up to a maximum of 5 mm junctional zone between the central-necrotic and the remaining peripheral-vascularized tumor segments. Second, PET was used for definition of a *hypometabolic junctional zone* between the necrotic and the peripheral hypermetabolic tumor segment. A SUV of 3 defined the boundary since a steep increase at a SUV of around 3–4 was seen moving from the necrotic toward the peripheral hypervascularized tumor. This volume was then subtracted from the peripheral remaining hypermetabolic-vascularized tumor segment to create the so called *Bystander Tumor Volume (BTV)* containing a *hypovascularized* and *hypometabolic tumor segment* (SUV ≤ 3). No additional margins (neither CTV nor PTV) were applied to the BTV. The pathologic lymph nodes and metastases were not irradiated.

### Radiotherapy technique

Patients were immobilized using the BodyFix (Elekta AB, Stockholm, Sweden). SBRT plans were calculated using Monaco with the Monte Carlo algorithm. Dose prescription depended on tumor site and volume and was delivered with VMAT by VERSA HD (Elekta AB, Stockholm, Sweden) in 1–3 fractions each of 10–12 Gy to the 70% isodose-line. Among the patients treated with the single-fraction regimen, if after the first restaging at 1 month it was still possible to define BTV, an additional adaptive single 10Gy-fraction was delivered. With regard to the dose constraints for OAR, those reported in TG101 were used [13]. Before each treatment, cone-beam CT (XVI system, VERSA HD) was obtained to verify the isocenter.

### Mechanisms behind NTE

The immunohistochemistry was performed on the available tissue samples after surgery following *neoadjuvant SBRT-PATHY* using antibodies for apoptosis-inducing factor (AIF), CD3, CD4, CD8, CD20, CD56, CD14, CD15, and S100 protein [14] to explore for the activation and modifications within the tumor microenvironment.

### Follow-up

Toxicity was evaluated using the *CTCAE Criteria* [15]. The first assessment of the radiological response was performed at 1 month after SBRT-PATHY by using CT and/or PET-CT, followed by repeated scans at month 2 and then every 3 months. Based on *RECIST criteria,* response to treatment was defined as a 30% or greater regression of partially-treated bulky tumor (representing BE), as well as unirradiated metastases (representing AE).

All procedures performed in present study were in accordance with the ethical standards. All the patients signed the informed consent after understanding the benefits and side effects of the treatment before any medical act is performed. The informed consent highlighted the unconventional and experimental nature of the radiation therapy intervention and its deviation from the current standard of care. It also highlighted their potential to discontinue the treatment or further treatments at any time and to pursue other forms of therapy including chemotherapy whenever clinical indication arises and/or agreeable to the patient. Most patients declined to pursue systemic therapy and, if initiated, they were not included in the study to highlight unbiased AE effects. Present study has been registered by the local ethic committee under study number A 26/18.

## Results

### Patient characteristics

**Fig. 3:**
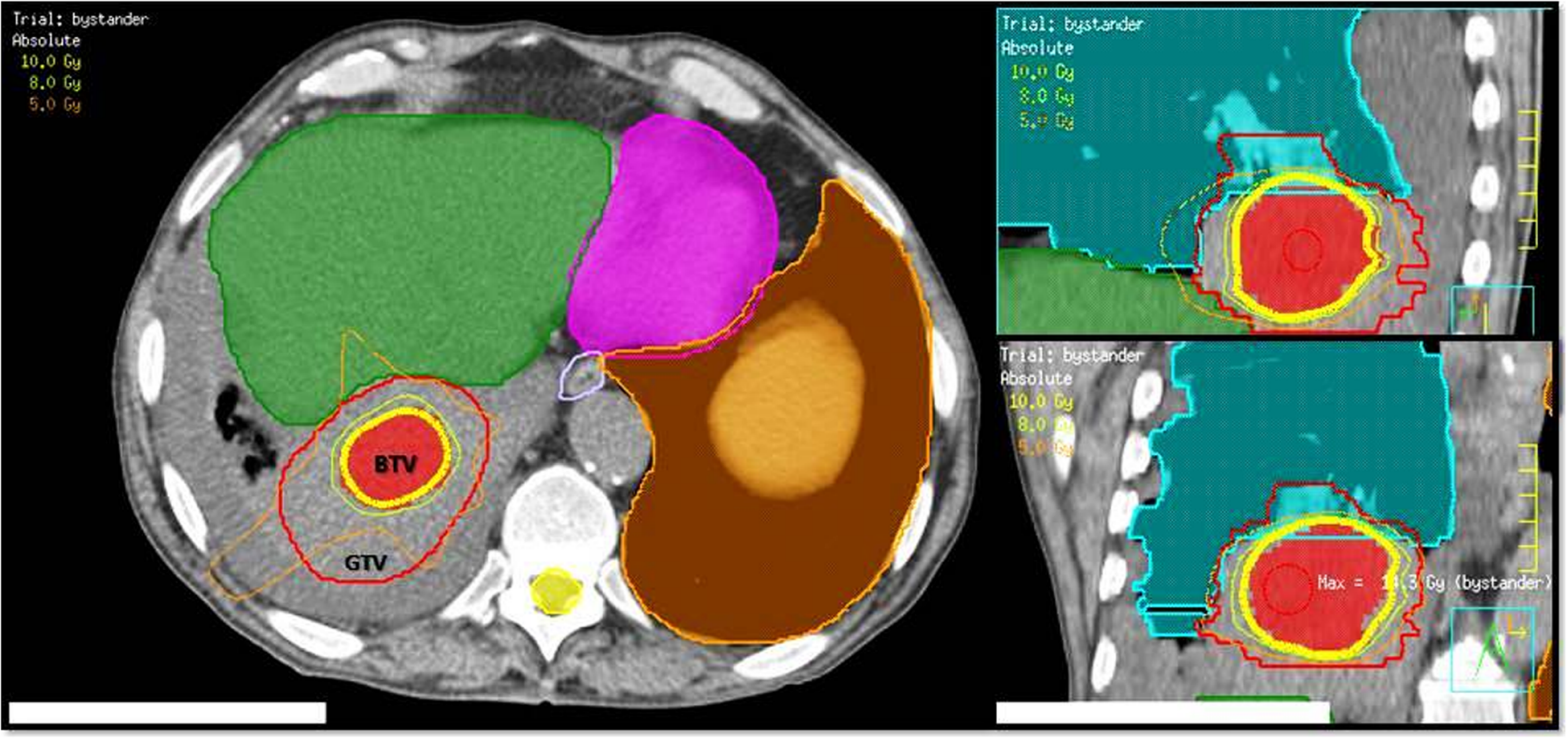
SBRT-PATHY DOSE DISTRIBUTION: Figure shows a large lung bulky tumor (GTV, red contour) irradiated partially by targeting exclusively the BTV (Bystander Tumor Volume-hypoxic segment) with 10 Gy (yellow isodose-line) in single fraction to the 70%-isodose line (Dma× 14.3 Gy). Green and orange isodose-lines correspond to 8 Gy and 5 Gy, respectively

Twenty-three patients, whose only bulky tumors were partially irradiated between March 2016 and February 2018, were included in this study. The median patient age was 74 years (range 41–86 y). The median bulky diameter was 8.8 cm (range 6–17 cm). The mean BTV volume was 57.1 mL (range 5–137 mL) corresponding to 32% of mean bulky volume of 179.8 mL (range 30–499 mL) (Table 1). Sixty-one percent of the patients had oligometastases (≤5 metastases in 1 organ, or ≤ 3 metastases in > 1 organ), and only 26% received chemotherapy or immunotherapy at least 4 weeks before SBRT-PATHY but not concomitantly or afterward, and all of them had disease progression under their systemic treatments. Mostly, the prescribed highly conformal dose to the BTV was 10 Gy × 1 to 70% (Dma× 14.5 Gy) in 15 (65%) patients (Fig. 3). In seven of them (30% of total), an additional adaptive single 10Gy-fraction was delivered after the first restaging at 1 month.

### Clinical outcome

The median follow-up time was 9.4 months (range 4–20 months). The local (BE) and distant (AE) response rates were 96% (22/23 patients) and 52% (12/23 patients), respectively. After a mean time of 4 weeks, the median bulky shrinkage was 70% (range 30–100%) with four (17%) complete responses, and 50% (range 30–100%) for unirradiated metastases. AE were observed at metastatic sites in the lung and in the neck, mediastinal and retroperitoneal lymph nodes. AE occurred mostly in patients (9/12, 75%) whose bulky tumors shrank more than 50% (in those with ≤50%, the AE rate was 25%); probably due to a small sample size, this result was not statistically significant (*p* > 0.05, data not shown). BE and AE persisted at the last follow-up in 96% (21/22) and 83% (10/12) of patients, respectively.

**Fig. 4:**
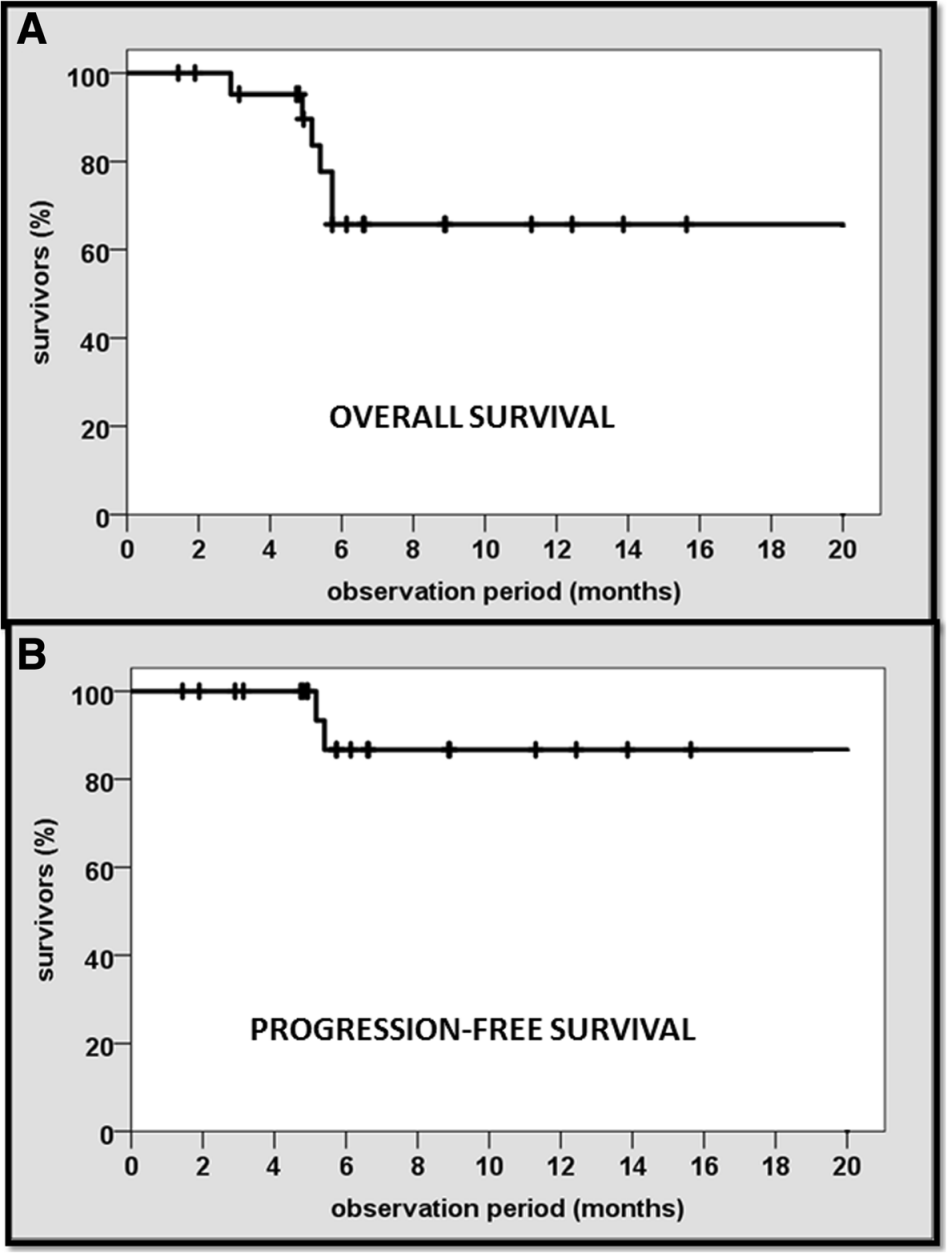
Actuarial Kaplan-Meier overall (**a**) and progression-free survival (**b**) of 23 patients

*Overall* and *progression-free survival rates were* 70% (16/23) and 87% (20/23), respectively (95% confidence interval) (Fig. 4a, b). Three patients died because of tumor progression. The mean time to tumor progression was 5 months. The overall response rate for symptom relief was 96% (22/23). After SBRT-PATHY, all patients drastically reduced or completely stopped taking analgesics. No patient experienced acute or late toxicity of any grade.

**Fig. 5:**
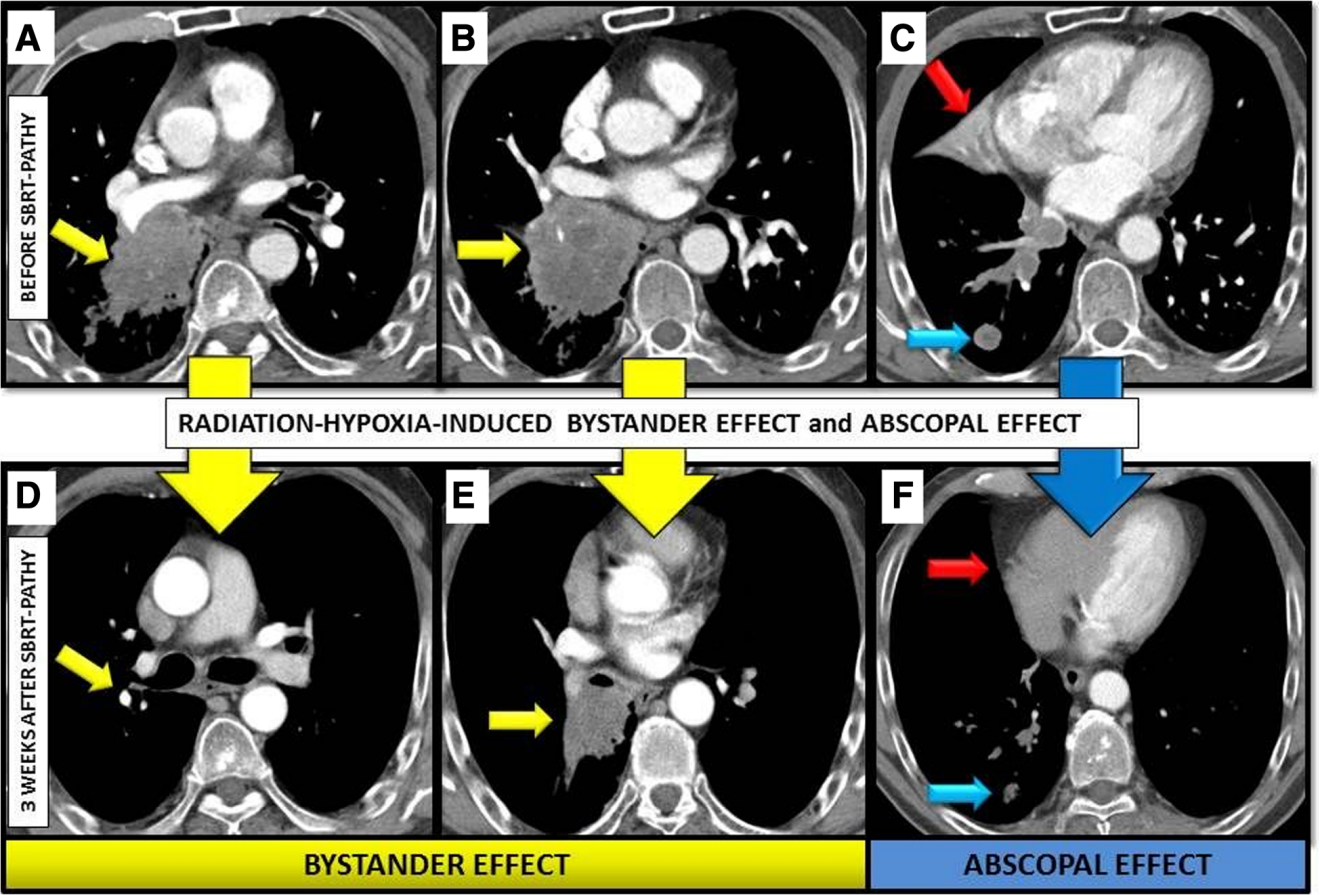
NEOADJUVANT SBRT-PATHY: **a-c**) diagnostic CT of the patient with unresectable squamous-cell lung cancer (yellow arrows), separate lung lesion (blue arrow), and an atelectasis (red arrow). 3 weeks after SBRT-PATHY, preoperative restaging CT **(d-f**) showed a 60% reduction of partially treated tumor (bystander effect: yellow arrows), a 50% reduction of unirradiated lung lesion (abscopal effect: blue arrows), and complete regression of atelectasis (red arrows)

One patient received *neoadjuvant exclusive SBRT-PATHY* for an un-resectable squamous-cell lung cancer (cT4, 8 cm): 10Gy × 3 to 70% (Dmax 43.5 Gy). In addition, he had a separate lower lobe lung lesion (2 cm) and metastatic mediastinal lymph nodes (cN2) that were not irradiated. After only 3 weeks, a preoperative CT scan showed 60, 50 and 30% reductions of partially irradiated bulky, un-irradiated separate lung lesion and metastatic lymph nodes respectively (Fig. 5). The remaining tumor was defined resectable and right bilobectomy with lymph node dissection was performed. Histological evaluation showed massive necrosis in the bulky tumor with a dense reaction at the border of the necrosis by infiltration of lymphocytes. Approximately 20% of the tumor cells were live (regression grade IIA sec. Junker [16]). A separate lung lesion was an 80% necrotic adenocarcinoma (IIA sec. Junker), but was showing no infiltrating lymphocytes on the borders of the tumor. Three metastatic lymph nodes were completely necrotic (regression grade III sec. Junker): ypT2a ypN0 (0/15) R0, stage yIB.

### Immunohistochemistry

AIF was massively upregulated in the carcinoma cells at all three tumor sites. CD20+ B-lymphocytes showed focal aggregates around the bulky tumor, a few were seen within the lymph node metastases, but were absent in the separate lung adenocarcinoma. CD3+ T-lymphocytes densely infiltrated the bulky tumor and were prevalent in the lymph node metastases, but were scarce in the adenocarcinoma. These T-lymphocytes were predominantly CD8+ cytotoxic lymphocytes, whereas CD4+ T-lymphocytes were scarce in the adenocarcinoma, and lymph node metastases, were mainly absent in the bulky tumor. CD56+ NK cells were not present at all three tumor sites. S100 protein antibodies predominantly stained macrophages at all three tumor sites. Myeloid-derived suppressor cells were predominantly stained by CD14 and were numerous at the bulky and lymph node tumor sites, but were less intense in adenocarcinoma. CD15+ myeloid-derived suppressor cells were seen in small numbers at all three tumor sites.

## Discussion

**Fig. 6:**
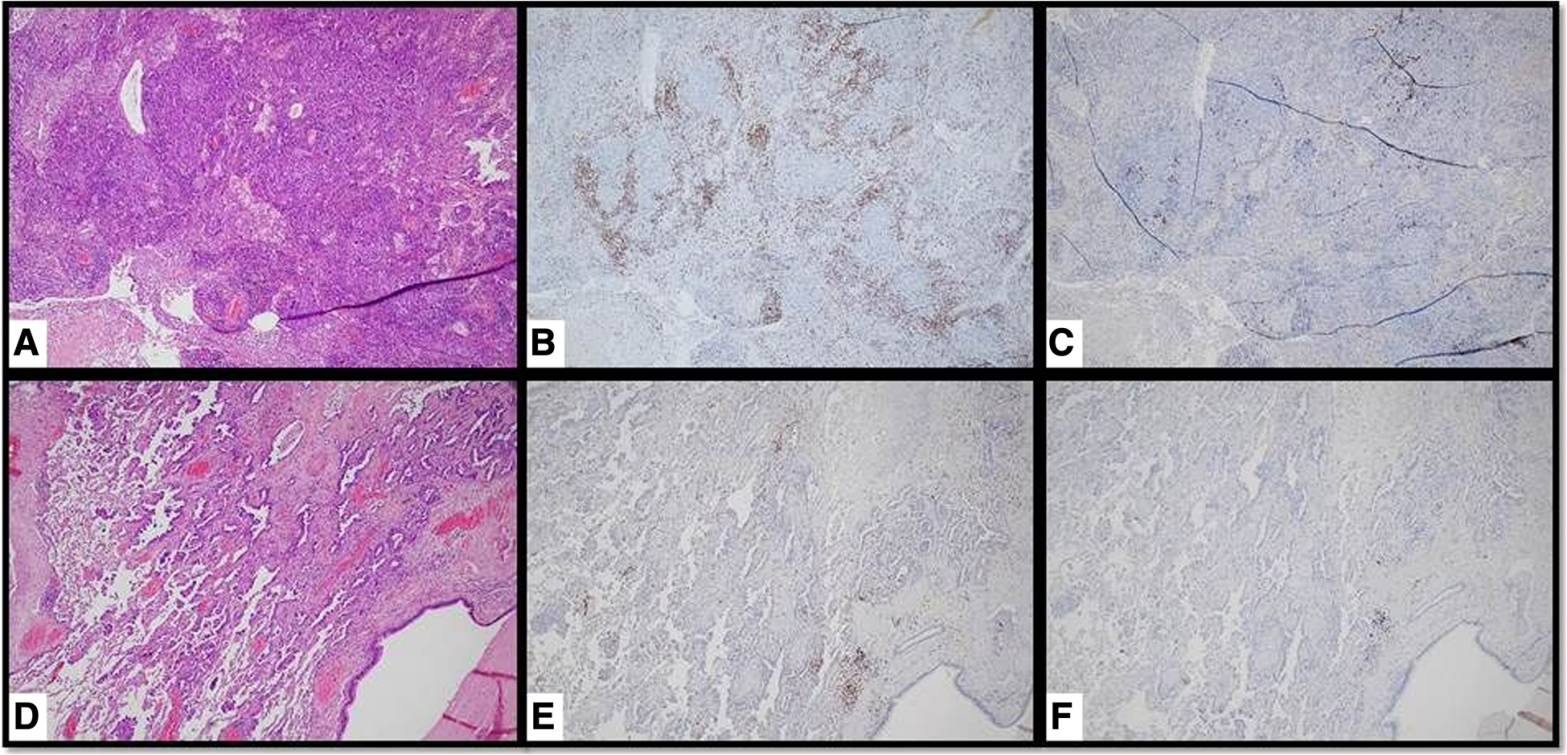
DIFFERENT LYMPHOCYTE PRESENTATION IN SQUAMOUS-CELL CARCINOMA (**a-c**) AND ADENOCARCINOMA (**d-f**): In squamous-cell carcinoma dense CD3+ T-lymphocyte infiltrates are present (**b**), which are scarce in adenocarcinoma (**e**). Some CD20+ B-lymphocytes are present in squamous cell carcinoma (**c**), but completely absent in adenocarcinoma (**f**). (**a** and **d**- Hematoxylin and Eosin staining; **b**, **c**, **e, f**- Immunohistochemistry; objective × 10)

**Fig. 7:**
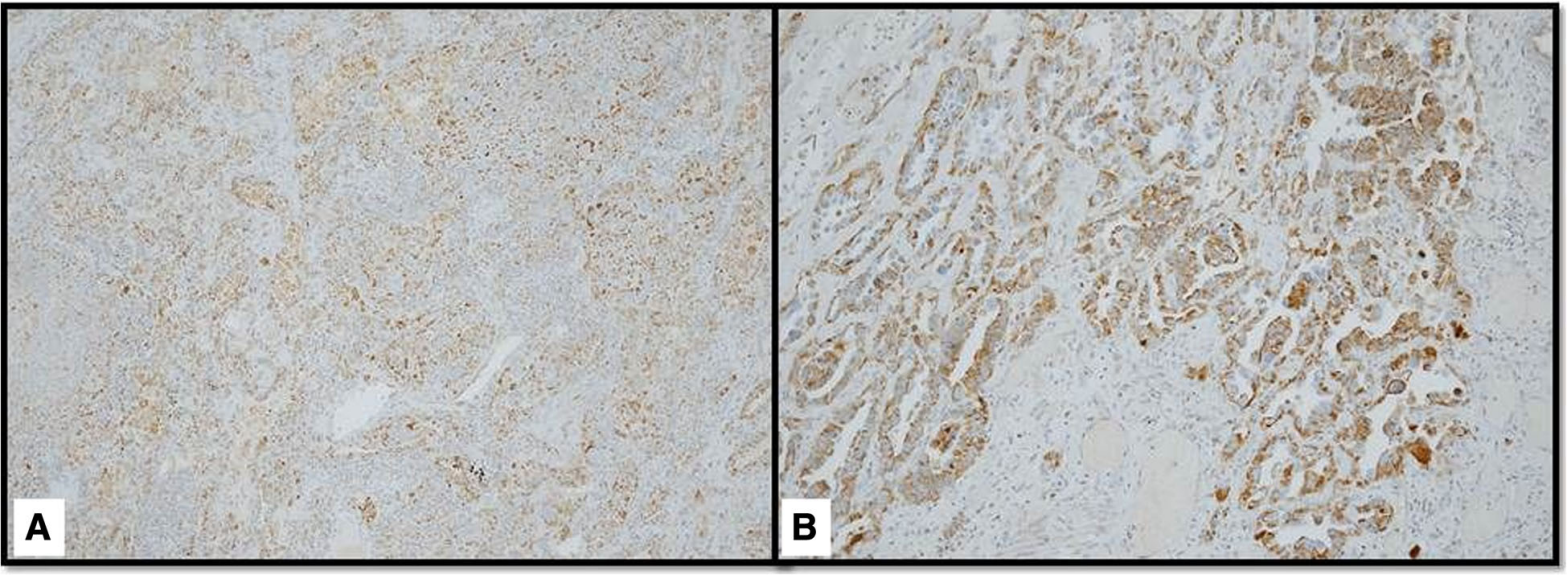
HISTOLOGICAL PRESENTATION OF APOPTOSIS-INDUCING FACTOR EXPRESSION: in squamous cell carcinoma **(a)** and adenocarcinoma **(b)**. (Immunohistochemistry, objective × 10)

Despite the advances in oncology, patients with bulky disease have worse prognosis compared with those with non-bulky tumors. *Higher tumor volume*, as a well-known independent adverse prognostic factor for local and regional recurrences, distant metastases, overall survival, and toxicity-related death, is associated with an increased clonal radioresistance and increased tumor hypoxia [1–5]. Bulky disease represents an important challenging obstacle for all currently available treatment options: surgery and conventional radiotherapy in most of the cases cannot achieve more than a palliative effect; SBRT is not indicated for such large tumors; whereas multimodality radio-chemotherapy options would induce higher toxicity. If in addition to bulky tumor, patients have metastases, thus complicating clinical situation, which is usually managed palliatively. SBRT-PATHY was purposefully developed with an aim to overcome previously mentioned bulky-related limiting factors, trying to improve the outcome of radiotherapy. The exploitation of BE and AE is what makes SBRT-PATHY an effective treatment. This technique was developed as a product of translation of our preclinical findings to a clinical setting [7], showing that high-dose irradiation of the hypoxic tumor selectively was able to generate stronger *non-targeted tumor killing* and *proliferative block* compared with those generated by the normoxic tumor. To the best of our knowledge, this is first and unique report on *induction of NTE in hypoxic conditions* by *partial tumor irradiation*, “turning” the tumor hypoxia into an ally in the fight against tumors. In addition to direct tumor killing, by the use of high-dose irradiation, SBRT-PATHY exploits the existing natural *anti-tumor immunity* and “*self-destructive*” (opposite to metastatic process) *abscopal tumor signaling* [7], boosting those available anti-tumor mechanisms. Even if underlying biological mechanisms are uncertain, they are not related exclusively to logarithmic cell killing. SBRT-PATHY overcomes limiting bulky volume by irradiating much smaller central hypoxic volume, which, in our series, makes up on an average only 32% of the entire bulky volume. Targeting the central third of the bulky tumor, mostly with single-fraction 10Gy, but inducing a median 70% bulky volume reduction, including almost 20% of complete responses, it reflects the potential that this approach offers. The problem related to the radio-resistant tumor hypoxia is addressed by the high-dose irradiation of this segment (*targeted-direct damage*) and induction of anti-tumor BE and AE (*non-targeted-indirect damage*). It is easy to assume that non-exposure of the tumor microenvironment to high-dose radiation due to a partial bulky irradiation (which is not the case with conventional radiotherapy) probably improves the effectiveness of the carriers of anti-tumor activity, which being very sensitive to radiation, would otherwise probably be killed with the conventional whole-tumor (+ CTV + PTV) irradiation. The presented results of this study support this hypothesis. There are evidences showing that the immune system activation, as a frequent lymphocyte infiltration within the irradiated tumor sites, can be associated with a favorable clinical outcome and survival [17–24]. The immunohistochemical findings after neo-adjuvant SBRT-PATHY are consistent with those evidences showing a dense immune reaction at the border of the bulky tumor. Abundant infiltration of the CD8+ T-lymphocytes was observed, indicating a possible anti-tumor-directed-activation of the immune system (Fig. 6a-c). In addition, AIF was massively upregulated in the partially irradiated bulky tumor, but also in the un-irradiated lung adenocarcinoma and mediastinal lymph node metastases (Fig. 7a, b). This would appear to indicate a significant apoptosis-induction, also at un-irradiated tumor sites. As AIF is a mitochondrial protein related to the cytochrome C apoptosis pathway, it would suggest alternative pathway activation [25]. The upstream gene for this activation needs to be studied further. Since there was significant lymphocyte infiltration at the bulky tumor, the same signs of immune system activation at abscopal site of the lung adenocarcinoma were clearly absent (Fig. 6 D-F). This is the first report of this kind to date considering the literature review on the abscopal responses [26], which were evaluated radiologically, but not directly by histological evaluation of the “abscopal tumor tissues”. It is believed that the abscopal responses were induced by the immune system activation at the abscopal sites which should be immunohistochemically demonstrable, but wasn’t actually clinically proven. With the limitations of our study, relative to the single case studied only with immunohistochemistry, our observation of such a massive necroptosis at an abscopal site, but completely devoid of the signs of immune system activation, brings us closer to our previous hypothesis that in the abscopal process in addition to the immune system, an important role is played by *abscopal tumor-signaling* [7, 12]. Amongst other things, this is also the first report that witnesses the existence of a *crossed radiation-induced abscopal effect*, observed after regression of an un-irradiated *adenocarcinoma*, following irradiation of the *squamous-cell carcinoma*. The further investigation on the induced abscopal mechanisms behind SBRT-PATHY are object of our ongoing prospective study.

Relative to the radiotherapy for bulky tumors, the current literature can provide us with retrospective outcomes on the use of two different unconventional approaches known as *spatially fractionated GRID radiotherapy* (SFGRT) [27] and *lattice radiotherapy* (LRT) [28]. SFGRT was used in the orthovoltage era to make possible the delivery of an effective radiation dose to deep-seated tumors, avoiding unacceptable skin toxicity [29]. Since the 1990s, SFGRT is used for the treatment of bulky tumors, delivering 10–15 Gy × 1 with multiple beamlets [30]. Several studies combined SFGRT and conventional radio-chemotherapy, confirming its efficacy and safety, reporting overall response rates of 78 to 91% without a notable increase in toxicity compared with conventional treatments (grade 3–4: 5.1%) [31–33]. LRT described by Wu [28, 34] is a novel technique that extrapolates bi-dimensional SFGRT to advanced three-dimensional radiotherapy whereby a high radiation dose, concentrated in small spheres called vertices, is located inside the tumor to keep a conventional dose in the tumor periphery, thus not affecting the tolerance dose of OAR. High effectiveness with low-grade toxicity of LRT is proven exclusively through single case reports [35, 36]. Compared with those techniques, SBRT-PATHY not combined with radio-chemotherapy or systemic therapy showed a higher overall response rate (96%) devoid of toxicity (grade 1–4: 0%). In addition to a high local response rate, 52% of our patients presented AE, thus representing a series with the highest AE rate reported in the literature. Previously, Golden [37] reported abscopal responses in 27% of patients after concomitant administration of GM-CSF and radiotherapy (3.5 Gy × 10). Considering the rest of the literature, only single case reports on unintentionally, occasionally induced AE, can be found [38]. The reason for such a high BE/AE rates in our series could probably be explained by the “*sparing effect*” to the tumor microenvironment from SBRT-PATHY, and a higher amount of released stimulating tumor antigens because of such a high bulky volume.

Even if the results of this study sound attractive, their nature is retrospective, and they are the product of a small series size that was not optimally homogeneous and had a short follow-up. At the time of the treatments planning, we did not have hypoxia-specific PET tracers. The definition of BTV combining 18F-FDG-PET and contrast-CT is not the most robust way to define the hypoxic segment, even if is capable of defining more aggressive tumor types correlated with HIF-1a expression in patients with gastric [39], tongue [40], NSCLC [41, 42], and oral squamous cell carcinoma [40–43]. However, our BTV corresponded to the central tumor region outside the contrast-enhanced tumor segment, which matched the results obtained by immunohistochemical staining of hypoxic cells using certain 2-nitroimidazoles, which typically showed increasing signal intensity as a function of increasing distance from the microvessel-carrying stroma [44]. Even if we cannot claim that BTV encompasses only and exclusively the hypoxic segment, we argue that it structurally corresponds to a hypo-vascularized tumor segment with very low metabolic activity. The definition of the BTV by using the hypoxia-specific PET tracers is the objective of our ongoing prospective study.

## Conclusions

When interpreting the results of the present study, its retrospective nature, small sample size, and short follow-up should be considered, requiring confirmation through a prospective trial. However, because this treatment is very safe and has an average bulky reduction of 70%, SBRT-PATHY showed high ***neoadjuvant*** potential to convert unresectable into resectable lesions (palliative into potentially curative treatments), and could be an option to consider for patients with bulky disease. As a treatment with high clinical response rates devoid of toxicity, it also has potential to improve the therapeutic ratio. It could be very convenient ***palliative*** 1-day treatment for patients in worse general conditions, offering an improved cost-effectiveness profile. Considering the very low radiation dose delivered outside the partially treated tumor, SBRT-PATHY could represent a very safe option as a ***salvage*** re-irradiation in case of relapses after previous radiotherapy, or as a neoadjuvant option before definitive radio-chemotherapy.

## Abbreviations

AE: Abscopal effect
AIF: Apoptosis-inducing factor
BE: Bystander effect
BTV: Bystander tumor volume
Flt-1: Fms-like tyrosine kinase
LRT: Lattice radiotherapy
NTE: Non-targeted effects
R-H-IAE: Radiation-hypoxia-induced abscopal effect
R-H-IBE: Radiation-hypoxia-induced bystander effect
SBRT: Stereotactic body radiation therapy
SBRT-PATHY: Stereotactic body radiation therapy for PArtial Tumor irradiation of unresectable bulky tumors targeting exclusively their HYpoxic segment
SFGRT: Spatially fractionated GRID radiotherapy
